# A Coordinated Analysis Examining the Association Between Personality Traits and Cognitive Dispersion

**DOI:** 10.1093/geroni/igab046.3237

**Published:** 2021-12-17

**Authors:** Tomiko Yoneda, Alejandra Marroig, Emily Willroth, Eileen Graham, Scott Hofer, Daniel Mroczek, Graciela Muniz-Terrera

**Affiliations:** 1 University of Victoria, Victoria, British Columbia, Canada; 2 Universidad de la República, Uruguay, Instituto de Estadística, Universidad de la República, Montevideo, Uruguay; 3 Northwestern University, Chicago, Illinois, United States; 4 University of Edinburgh, Edinburgh, Scotland, United Kingdom

## Abstract

Cognitive dispersion is the degree of within-person variation in performance across cognitive tasks at the same testing occasion. Existing literature indicates that cognitive dispersion may be an early marker of poor brain health, dementia and mortality. Limited research, however, has examined individual differences in cognitive dispersion. Although personality traits are associated with individual differences in cognitive functioning, no research has examined personality and cognitive dispersion. In this project, we execute a pre-registered, coordinated analysis of seven diverse, international longitudinal studies of aging (Ntotal=33,581; mean age range=56.4-71.2) to investigate the extent to which the Big Five personality traits are associated with cognitive dispersion. For methodological approach, see /osf.io/wrnjq/. Cognitive dispersion scores were derived from cognitive test results, and independent linear regression models were fit independently in each study to examine personality traits as predictors of dispersion scores, adjusting for mean cognitive performance and socio-demographics (age, sex, education). Results from individual studies were synthesized using random-effects meta-analyses. Results revealed minimal evidence for associations between cognitive dispersion and personality traits in independent analyses or in meta-analyses. Based on the meta-analytic estimates, only higher levels of openness were associated with greater cognitive dispersion. Mean cognitive scores were negatively associated with cognitive dispersion across the majority of studies, indicating that individuals with higher mean performance had less dispersed cognitive scores. Our study contributes to the replicability and transparency efforts characteristic of open science by pre-registering our study and drawing on the collaborative network of the Integrative Analysis of Longitudinal Studies of Aging and Dementia (IALSA).

